# Early Oral Nutritional Supplements in the Prevention of Wheezing, Asthma, and Respiratory Infections

**DOI:** 10.3389/fped.2022.866868

**Published:** 2022-03-25

**Authors:** Anna Trivillin, Sara Zanella, Raimondo Junior Castaldo, Francesco Prati, Stefania Zanconato, Silvia Carraro, Valentina Agnese Ferraro

**Affiliations:** Department of Women's and Children's Health, University of Padua, Padua, Italy

**Keywords:** wheezing, pediatric asthma, respiratory tract infection (RTI), prebiotics and probiotics, vitamin D, fish, PUFA, children

## Abstract

Wheezing, asthma, and respiratory infections (RTI) are among the most common causes of morbidity in children and their economic and social burden could be significantly reduced by specific prevention strategies. Epidemiological studies suggest that lower levels of some nutrients are associated with higher prevalence of these conditions, but the possible protective effect of early supplementation with these nutrients has not yet been established. Aim of our review is to synthetize the available scientific evidence on the role of supplementation with pre- and probiotics, vitamin D, fish and poly-unsaturated fatty acids (PUFA), vitamin A, C, and E, given during the first year of life, in the prevention of wheezing, asthma and RTI. We searched studies published on this topic in the PubMed database between January 2000 and September 2021. As for pre- and probiotics, most of the studies showed that an early supplementation had no protective effect toward the development of asthma and wheezing, while conflicting results were reported on their role in the reduction of RTI. As for vitamin D, the available data suggest that early and regular (on a daily or weekly base) supplementation of vitamin D during infancy could have a role in the prevention of RTI, while most studies showed no effect in the prevention of wheezing or asthma. Finally, early introduction of fish in the diet in most studies has proved protective toward wheezing and asthma development.

## Introduction

In recent decades the worldwide prevalence of allergic and respiratory diseases has noticeably increased, and these diseases have become a real burden for the healthcare system and society (1). Current evidence suggests that these conditions have a multifactorial etiology resulting from the interaction between genetic susceptibility, host-related factors and environmental exposure (1, 2).

Among environmental factors, diet has a high impact on individual respiratory health by regulating the immune system and nutrition during the first years of life and it is critical for enhancing health outcomes (3). Several studies have investigated the potential benefit of supplementing nutrients in early infancy for reducing the risk of developing allergic and respiratory disease during childhood (3, 4). Among the nutritional supplements available for oral administration in children, prebiotics and probiotics, vitamin D, fish and Poly-Unsatured Fatty Acids (PUFA), vitamin A, C, and E are the better studied.

Aim of our narrative review is to synthetize the available scientific evidence on the role of supplementation of these nutrients during the first year of life, in the prevention of wheezing, asthma, and respiratory tract infections (RTI) during childhood, in order to understand their potential benefit and thus supporting or discouraging their use.

## Methods

We searched published studies in the PubMed database by combining the following terms: “probiotic,” “prebiotics,” “vitamin D,” “fish,” “PUFA,” “vitamin A,” “vitamin C,” “vitamin E” as nutritional supplements, and “asthma,” “wheeze,” “respiratory infections” as outcomes.

The search strategy included filters for language (English), age of study subjects (infants and children), and year of publication (we included papers published between January 2000 and September 2021).

Finally, we only included studies that considered nutrients exposure through diet or specific nutrient supplementations in the first year of life. Studies that evaluated only nutrient's serum levels or nutrient's supplementations given only to mothers during pregnancy and/or lactation were excluded.

## Prebiotics and Probiotics

Probiotics are live microorganisms which confer a health benefit when administered in adequate amounts (5). Prebiotics are dietary substances (mostly polysaccharides and oligosaccharides poorly digested by human enzymes) that favor the growth of selected beneficial bacteria living in the gut (5). Synbiotics are loosely defined as mixtures of pre- and probiotics that beneficially affect the host (6).

The idea that pre- and probiotics could play a role in reducing the risk of allergic disease come from the hygiene hypothesis which states that early childhood exposure to some microorganisms protects against allergic diseases by contributing to the development of the immune system. In particular, a lack of exposure is thought to impair the development of immune tolerance. Moreover, because of a potential competitive role against resident flora and because of their immunomodulating effect, early supplementation with pre- and probiotics has been studied as possible tool to prevent RTI in children (7).

Several studies, including randomized clinical trials (RCTs), have investigated the potential benefit of various strains and forms of pre- and probiotics in decreasing the risk of asthma, wheezing, and recurrent RTI, but no clear recommendations are nowadays available about their use in children.

### Prebiotics and Probiotics in Prevention of Asthma and Wheezing

Fifteen trials (8–22) (Table 1) investigated the role of supplementation with pre- and probiotics in the prevention of asthma and wheeze. In these studies, different strains of probiotics were used, the most common being *Lactobacillus rhamnosus* and *Bifidobacterium breve*. The supplementation was usually started in the mothers during the last gestational weeks (35-36 gw) and it was continued in offspring. Most of these trials were conducted in children at risk for allergic diseases (i.e., first-degree relatives with asthma or allergic diseases).

**Table 1:** Main features and results of studies investigating active supplementation of pre- and probiotics.

**References**	**Study design**	**Population**	**Conclusion**
**Pre- and probiotics and asthma/wheezing**			
(9)	Randomized, double-blind controlled trial	184 high-risk infants supplemented in the first 6 months of life	Early LGG supplementation does not prevent the development of asthma at 2 years of age
(10)	Randomized double-blind controlled trial	253 infants at risk for allergy, supplemented in the first 6 months of life	At the age of 5 years, in children who had developed asthma, there were no significant differences between the groups supplemented or not
(11)	Randomized double-blind controlled trial	231 newborns of women with allergy, supplemented for the first 6 months of life	The rate of wheezing was significantly higher in the probiotic group in the second 6 months of life
(12)	Randomized double-blind controlled trial	153 newborns of women with allergy, supplemented in the first 6 months of life	No differences in probiotic and control group in the rate of asthma at 2.5 years
(13)	Randomized double-blind controlled trial	178 infants with atopic mothers, supplemented in the first 6 months of life	No differences in probiotic and control group in the rate of asthma at 5 years
(14)	Randomized controlled trial	75 infants with atopic dermatitis, supplemented in the first 6 months of life	The prevalence of frequent wheezing and the number of children treated with asthma medication was significantly lower in the synbiotic than in the placebo group
(15)	Randomized double blind controlled trial	171 children <13 months of life, supplemented from the 4th to the 13th month of life	No long-term effect in infants supplemented with probiotics on development of asthma
(16)	Randomized controlled trial	1,223 mother-infant pairs, supplemented from 36gw (mothers) to 6 months of life (infants)	The lifetime prevalence of asthma was similar in the probiotic and placebo groups
(17)	Randomized double blind controlled trial	232 mother-infant pairs, supplemented from 36gw (mothers) to 12 months of life (infants)	No differences in probiotic group and control group in the rate of wheeze at 2 years
(18)	Randomized double blind controlled trial	1,223 mother-infant pairs, supplemented from 36gw (mothers) to 6 months of life (infants)	No differences in probiotic and control group in the rate of asthma at 5 years
(19)	Randomized double blind controlled trial	131 mother-infant pairs, supplemented from 36gw (mothers) to 6 months of li**f**e (infants)	No differences in probiotic and control group in the rate of asthma at 5 years
(20)	Randomized controlled trial	425 mother-infant pairs, supplemented from 35gw (mothers) to 6 months of li**f**e (infants)	No differences in probiotic and control group in the rate of asthma at 4 years
(8)	Randomized double blind controlled trial	1,223 mother-infant pairs, supplemented from 36gw (mothers) to 6 months of li**f**e (infants)	No differences in probiotic and control group in the rate of asthma at 13 years
(21)	Randomized double blind controlled trial	171 infants, supplemented in the first 6 months of life	No differences in probiotic and control group in the rate of wheezing
(22)	Randomized controlled trial	83 pregnant women with a positive family history of allergic disease, supplemented during pregnancy (mothers) to the first year of life (infants)	No differences in probiotic and control group in the rate of asthma
**Pre- and probiotics and RTI**			
(23)	Randomized double blind controlled trial	203 children, aged 6-36 months, supplemented for 90 days	Use of the probiotic strains BB-12 and L3 statistically reduced the risk of URTIs in healthy children
(24)	Randomized, double-blind controlled trial	188 infants, supplemented from 6 to 16 months of life	Administration of a formula with probiotics may be useful for the prevention of community-acquired and upper RTI
(25)	Randomized, double-blind controlled trial	81 infants, supplemented for the first 12 months of life	Probiotics may offer a safe means of reducing the risk of early acute otitis media and antibiotic use and the risk of recurrent RTI during the first year of life
(26)	Randomized, double-blind controlled trial	109 infants, supplemented for the first year of life	The infants receiving BB-12 were reported to have experienced fewer RTI than the control infants
(27)	Randomized, double-blind controlled trial	109 infants, supplemented for the first 2 years of life	The infants receiving BB-12 were reported to have experienced fewer RTI than the control infants
(28)	Randomized, double-blind controlled trial	224 children, supplemented from 7 to 13 months of life	The pro- and prebiotics included in follow-up formula do not reduce the risk of AOM, recurrent AOM, antibiotic use or lower RTI at 1 year
(29)	Randomized, double-blind controlled trial	43 infants, aged 4-46 months, supplemented for 4 months	No significant differences regarding the number of episodes of AOM in the active group and in the placebo group
(30)	Randomized, double-blind controlled trial	201 infants, aged 4-10 months of life, supplemented for 12 weeks	Rate and duration of respiratory illnesses did not differ significantly between groups

Kallio et al. (8) published the largest cohort study on the effect of a mixture of probiotics given from the 36 gw to 1,223 women carrying a child at high risk for allergy and then to the offspring up to the age of 6 months. At the 13-year follow-up, the prevalence of doctor diagnosed allergic diseases, including asthma, was not statistically different in children compared to controls.

Likewise, the vast majority of the available trials (Table 1) showed no protective effect of early pre- and probiotics supplementation with respect to the development of asthma and wheezing.

### Prebiotics and Probiotics in the Prevention of RTI

Eight trials (Table 1) investigated the role of early supplementation of pre- and probiotics in the prevention of RTI. In these studies, the most commonly used probiotics were *Lactobacillus rhamnosus* and *Bifidobacterium Lactis*. Supplementation was usually started in the first months of life and it was continued for a variable period, ranging from 12 to 46 months.

The largest sample size was analyzed by DI Pierro et al. (23), who recruited 203 children (aged 6-36 months) demonstrating that the supplementation with a probiotic mixture (containing *Bifidobacterium animalis* subspecies lactis BB-12 and *Enterococcus faecium* L3) was associated with a 84% reduction of RTI episode rate and a 50% reduction of their duration.

Nonetheless, when taken altogether, the available trials provide conflicting results: in five trials (23–27) administration of pre- and/or probiotics was associated with a reduction in the prevalence of RTI, while in three trials (28–30) no significant difference was reported in children treated with pre- and/or probiotics.

## Vitamin D

Vitamin D is a fat-soluble molecule mostly derived from conversion of 7-dehydrocholesterol when the skin is exposed to ultraviolet irradiation and partly derived from the diet (31).

Several studies have demonstrated that vitamin D has important biologic activities on the innate and adaptive immune systems and it can play a role in the onset and progression of immune-related diseases (31).

Here, we synthetize the available scientific literature on the role of Vitamin D supplementation in infants, to clarify whether it has a role in the prevention of wheezing, asthma, and RTI during childhood.

### Vitamin D in Prevention of Wheezing and Asthma

The efficacy of vitamin D supplementation in the first year of life in the prevention of wheezing and asthma is still debated. According to our research strategy, we found six studies (Table 2) that analyzed the outcome wheezing and 5 the outcome asthma.

**Table 2:** Main features and results of studies investigating active supplementation of Vitamin D.

**References**	**Study design**	**Population**	**Conclusion**
**Vitamin D and wheezing**			
(32)	Randomized double-blind controlled trial	195 infants, supplemented with Vitamin D (400 IU/d) or placebo from birth until 6 months of age. Some infants wore personal UV dosimeters to measure direct UV light exposure	The frequency of wheezing did not differ significantly between the vitamin D and placebo group No significant difference in UV exposure between infants who did or did not have doctor-diagnosed wheeze in the first 6 months of life
(33)	Prospective cohort study	5161 children (0-5 years) supplemented with single product vitamin D, multivitamin or multivitamin with iron, containing usually a vitamin D dose of 400 IU	Vitamin D supplementation during pregnancy was associated with reduced wheezing, but child vitamin D supplementation was not associated with reduced wheezing
(34)	Randomized controlled trial	975 infants supplemented with 400 IU/day or 1,200 IU/day from the age of 2 weeks. At 12 months of age, wheezing was evaluated	The number of children with wheezing was similar in both groups. High-dose vitamin D supplementation did not prevent wheezing during the first year of life
(35)	Randomized double-blind controlled trial	195 infants at “high-risk” for allergic disease (positive family history) and born from mothers with a sufficient 25(OH)D serum concentration between 36 and 40 GW. Oral vitamin D supplementation (400 IU/day) or a placebo for the first 6 months of life	No statistically significant differences in the incidence of allergic diseases (including wheezing) over the first 2.5 years of life between the vitamin D and placebo group
(36)	Prospective cohort study	300 premature infants (28-34 gw), supplemented with multivitamin, containing 400 IU/dose of cholecalciferol, in first year of age	Black infants supplemented with multivitamins early in life experienced increased wheezing, whereas non-black supplemented infants experienced decreased wheezing
(37)	Randomized double-blind controlled trial	300 black preterm infants, supplemented for the first 6 months of age with 400 IU of cholecalciferol or placebo	Among black infants born preterm, sustained supplementation with vitamin D, compared with diet-limited supplementation, resulted in a reduced risk of recurrent wheezing by 12 month of age
**Vitamin D and asthma**			
(35)	Randomized double-blind controlled trial	195 infants at “high-risk” for allergic disease (positive family history) and born from mothers with a sufficient 25(OH)D serum concentration between 36 and 40 GW. Oral vitamin D supplementation (400 IU/day) or a placebo for the first 6 months of life	No statistically significant differences in the incidence of allergic diseases (including asthma) over the first 2.5 years of life between the vitamin D and placebo group
(38)	Population-based cohort	61,676 infants, supplemented in the first 6 months of life with vitamin D only, cod liver oil, multivitamins, and any vitamin D supplement	No protective effect of vitamin D only, or cod liver oil, on asthma at school age
(39)	Population-based cohort	8,690 infants, supplemented in the first year of life (with the contemporary recommended dose of 2,000 UI/day)	Vitamin D supplementation in the first year of life is associated with an increased risk of asthma later in life (at 31 years of age)
(40)	Randomized double-blind controlled trial	260 mother-infant pairs, supplemented from birth to 6 months in one of the following groups: placebo/placebo, 1,000 IU/ 400 IU or 2,000 IU/800 IU	The number of primary care asthma visits per child and the number of visits per child for which salbutamol or prednisone was prescribed were smaller in the vitamin D supplemented groups
(41)	Prospective birth cohort	4,089 newborn infants, supplemented in the first year of life with vitamins based in peanut oil, in water-soluble form, in both the 2 preparations, and no vitamins	Children supplemented with vitamins A and D in water-soluble form during the first year of life had an almost 2-fold increased risk of asthma at age 4 years compared with those receiving vitamins in peanut oil
**Vitamin D and RTI**			
(42)	Randomized double-blind controlled trial	239 infants, supplemented from birth and for 9 months with 10 μg vitamin D preparation orally daily or placebo syrup	Daily infant supplementation with vitamin D along with sun exposure is superior to sun exposure alone in maintaining normal infant 25(OH)D at 3.5 months, and provide protection from infectious morbidity
(43)	Prospective birth cohort study	2,244 infants, supplemented with Vitamin D (daily dose 400-600 IU) from birth to 6 months of age	Infants supplemented with Vitamin D had a longer period without experiencing the first RTI and a decreased risk of RTI
(44)	Randomized double-blind controlled trial	3,460 infants aged 1–11 months, supplemented with oral 100,000 IU (2.5 mg) vitamin D3 or placebo, once every 3 months for 18 months	No significant difference between the incidence of pneumonia between the vitamin D and the placebo group

With regard to wheezing, a recent double-blind, placebo-controlled trial (32) on 195 infants demonstrated no statistically significant difference in wheezing frequency at 12 months of life in children supplemented with vitamin D in the first 6 months of life. Likewise, Anderson et al. (33) showed in a cohort study of 5,161 children (0–5 years) that vitamin D supplementation was not associated with reduced prevalence of wheezing. Moreover, a Finnish RCT (34) on 975 vitamin D-sufficient infants demonstrated that nor standard vitamin D supplementation (400 IU) neither higher vitamin D supplementation (1,200 IU), during the first year of life, decreased allergic diseases and wheezing evaluated at 12 months of age.

Among infants at “high-risk” for allergic diseases (one first-degree relative with asthma, eczema, or allergic rhinitis), Reuter et al. (35) in a double-blind RCT showed no statistically significant differences in incidence of any doctor-diagnosed allergic disease outcomes or allergen sensitization rates between the vitamin D-supplemented and placebo groups at either 1 or 2.5 years of age.

Among preterm black infants, Hibbs et al. published two contrasting study: the first one (36) showed that supplementation with multivitamins (containing 400 IU/dose of cholecalciferol) in the first year of life was associated with a prevalence of wheezing increased in preterm black infants and reduced in non-black infants; the second one (37) showed that in preterm black infants sustained supplementation with vitamin D (400 IU/d) compared with diet-limited supplementation (200 IU/d) was associated with a reduced risk of recurrent wheeze by 12 months' adjusted age.

With regard to asthma, a recent double-blind randomized controlled trial (35) and two previous cohort studies (38, 39) showed no efficacy of early vitamin D supplementation in the prevention of asthma. On the other hand, two other studies (40, 41) report a protective role of vitamin D given in the first year of life on reducing the risk of developing asthma during childhood. Recently, both a systematic review (45) and a document of the World Allergy Organization (46) found no support for the hypothesis that vitamin D supplementation in healthy term infants reduces the risk of developing asthma in childhood.

In conclusion, although some studies report a possible protective effect, most studies did not find a significant role of vitamin D supplementation during the first year of life in the prevention of wheezing and asthma.

### Vitamin D in Prevention of RTI

*In vitro* studies showed that vitamin D has a role in the prevention of both bacterial and also viral RTI, since it induces the production of antimicrobial peptides (47), and it reduces the inflammatory response to viral infections (48).

Considering studies conducted *in vivo*, a Cochrane (49) reported no benefit from vitamin D supplementation in children under 5 years of age in preventing pneumonia and tuberculosis, while a more recent individual data meta-analysis (including 11,321 participants, aged 0-95 years) (50) showed that vitamin D supplementation can prevent acute RTI with the greatest benefit in deficient subjects and in those supplemented daily or weekly.

According to our research strategy, we identified four studies (Table 2), which, indeed, differs for population, intervention, and outcomes. A double-blind, placebo-controlled trial conducted in India (42) showed that daily infant supplementation with vitamin D for 9 months after birth is superior to sun exposure alone in maintaining normal infant 25(OH)D, and provide protection from infectious morbidity. In keeping, Hong et al. (43) in a prospective birth cohort study (2,244 infants) demonstrated an inverse association between the frequency of vitamin D supplementation (400-600 IU/day) during the first 6 months of life and the risk of RTI, lower RTI, and RTI-related hospitalization. Also, Manaseki-Holland et al. (44) demonstrated that oral supplementation of vitamin D3 given to infants every 3 months for 18 months does not reduce the incidence of pneumonia.

Taken together, these studies suggest that early and regular (on a daily or weekly base) supplementation of vitamin D during infancy could have a role in the prevention of RTI.

## PUFA

Polyunsaturated fatty acids (PUFAs) are fatty acids characterized by more than one double bond along the hydrocarbon chain, which have a high nutritional value and some of them, such as omega-3 and omega-6, are essential. One of the main nutritional sources of polyunsaturated fatty acids is fish (51).

Fifteen articles are nowadays available on the role of active supplementation or early introduction of fish and PUFA in the first year of life in the prevention of wheezing, asthma and RTI. We summarize the articles in Table 3 and, given that the majority of them show the same conclusions, we added quantitative information about the degree of protection.

**Table 3:** Main features and results of studies investigating active supplementation of PUFA and early introduction of fish in diet.

**References**	**Study design**	**Population**	**Conclusion**
**Active supplementation of PUFA**			
(52)	Multicenter, prospective, observational study	1,342 infants, supplemented in the first year of life	Infants fed formula supplemented with DHA/ARA (DHA + group) had a lower incidence of bronchiolitis/bronchitis compared with control group at 5 months (6.1 vs. 13.9%, *p* = 0.0001), 7 months (5.1 vs. 10.8%, *p* = 0.01), and 9 months (5.8 vs. 11.3%, *p* = 0.01). The incidence of upper airway infections was lower in DHA + group compared with control group at 1 month (6.6 vs. 12.1%, *p* = 0.05) and 12 months (16.2 vs. 24.2%, *p* = 0.01).
(53)	Multicenter, prospective, observational study	325 infants, supplemented in the first year of life	Infants fed formula supplemented with DHA/ARA, compared with controls, had lower incidence of bronchitis/bronchiolitis (OR 0.41; 95% CI: 0.24-0.70; *p* = 0.001) and croup (OR 0.23; 95% CI: 0.05-0.97; *p* = 0.045)
(54)	Randomized double-blind controlled trial	147 infants, supplemented from 5 days to 12 months of life	Infants fed formula supplemented with DHA/ARA had a significantly lower odds of having an increased number of episodes of upper respiratory infections (OR 0.32; 95% CI 0.14-0.75; *p* = 0.008), wheezing/asthma (OR 0.31; 95% CI 0.10-0.90; *p* = 0.03), wheezing/asthma/atopic dermatitis (OR 0.29; 95% CI 0.12-0.72; *p* = 0.008), or any allergy (OR 0.30; 95% CI 0.12-0.73; *p* = 0.008) during the first 3 years of life compared with the control group
(55)	Randomized controlled trial	91 infants, supplemented in the first year of life	Infants fed formula supplemented with DHA/ARA had a lower incidence of asthma and wheezing in the first 4 years of life compared the control group (OR 0.57; 95% CI 0.2-1.6)
(56)	Multicenter controlled intervention study	6,154 infants, supplemented in the first 2 years of life	Infants with higher intake of *n* – 3 PUFAs and oily fish had no significant difference in the incidence of allergic disease and wheeze compared with control cohorts (OR adjusted 0.91; 95% CI 0.79-1.06)
(57)	Randomized double-blind controlled trial	420 infants at high-risk of atopic diseases, supplemented from birth to 6 months	No differences in prevalence of allergic outcomes between infants in the fish oil and control groups at 12 months (37.8 vs. 39.5%)
(58)	Randomized controlled trial	616 infants with a family history of asthma, supplemented from 6 months or at onset of bottle-feeding and during the first 5 years of life	In children with a family history of asthma dietary fatty acid modification do not reduce the prevalence of asthma [absolute rik reduction (ARR) −4.8; 95% CI −12.5-2.9], or other atopic disorders at age 8 years.
**Early introduction of fish in diet**			
(59)	Population based multiethnic prospective study	7,210 subjects Exposure in the first 14 months of life	Children who were given fish between 6 and 12 months had a lower risk of wheezing at 48 months (OR 0.64; 95% CI 0.43-0.94). When compared with introduction between 6 and 12 months, no introduction in the first year and introduction between 0 and 6 months were associated with an increased risk of wheezing at 48 months (OR 1.57; 95% CI 1.07–2.31 and OR 1.53; 95% CI 1.07-2.19, respectively).
(60)	Controlled, population-based, primary intervention trial	20,544 subjects Exposure in first 2 years of life	Eating fish at least once a week at one year of age was associated with a 40% and 34% reduction in the odds of asthma, and wheeze at 6 years of age
(61)	Prospective, longitudinal cohort study	4,171 subjects Exposure in the first year of life	The introduction of fish before the age of 9 months reduced the risk of recurrent wheeze (OR adjusted 0.6; 0.4-0.8).
(62)	Prospective cohort study	4,089 subjects Exposure in the first year of life	Children receiving fish between 3 and 8 months of age had a reduced risk for asthma at 4 years of age (OR adjusted 0.73, 95% CI 0.55-0.97).
(63)	Prospective cohort study	2,531 children Exposure in the first 12 months of life	Fish consumption in the first year of life is protective toward the development of asthma at the age of 4 years with adjusted OR 0.84 (95% CI = 0.57, 1.22).
(64)	Prospective cohort study	3,285 subjects Exposure in the first year of life	Children who consumed fish at 1 year of age had an overall reduced incidence of asthma up to the age of 12 (OR adjusted 0.80; 95% CI 0.65, 0.98; p = 0.034)
(65)	Double-blind controlled trial	738 subjects Exposure in the first 3 years of age	Inverse associations were seen between asthma and/or recurrent wheeze in 3-year-old children and dietary intakes of total PUFA (OR 0.65; 95% CI 0.38-1.09; p=0.10), omega-3 (OR 0.61; 95% CI 0.36-1.02; p=0.06), and omega-6 PUFA (OR 0.53; 95% CI 0.31-0.90; p=0.02), though this was statistically significant only for omega-6 PUFA intake.
(66)	Prospective cohort study	3,086 subjects Exposure in the first year of life	The association between children's consumption of fish at 1 year of age and asthma at 2 years was weak and insignificant. The binary logistic regression of the association between children's consumption of fish at 1 year of age and doctor-diagnosed asthma at 2 years showed any significant results (cod liver oil *p* = 0.43 and *p* = 0.54; any kind of fish *p* = 0.16; oily fish *p* = 0.86; lean fish *p* = 0.17; vegetables *p* = 0.42 and *p* = 0.86).

### Active Supplementation of PUFA

A multicenter prospective study on 1,342 infants showed that a formula milk enriched with DHA (Docosahexanoic acid) and ARA (arachidonic acid) can reduce the incidence of upper RTI, bronchitis and bronchiolitis (52). Similar results were reported in a more recent multicenter study on 325 infants (53). Furthermore, Birch et al. (54) in a double-blind RCT showed that infants fed for the first 12 months of life with a DHA- and ARA-enriched formula showed not only a reduced incidence of RTI but also of wheezing and asthma, compared to placebo. In keeping with this, a randomized control trial with a 4-year follow-up (55) showed that children fed for the first year of life with a formula supplemented with DHA/ARA have a reduced incidence of asthma and wheeze.

On the other side, a Norwegian multicenter controlled study on 6,154 infants, followed up to the age of 2 years, showed that neither the incidence of allergic disease nor the incidence of wheezing was statistically different in children who took a diet enriched of PUFA and oily fish compared to the control cohort (56). In keeping with this study, a double blind RCT on 420 infants at high atopic risk, fed from birth to 6 months with a diet enriched with DHA and EPA (Eicosapentaenoic Acid) or with a control diet, reported no differences in the prevalence of asthma and wheeze at 12 months of age (57). Furthermore, the RCT Childhood Asthma Prevention Study shows that, in children with a family history of asthma, the implementation of fatty acids in the diet of the first years of life had no effect on reducing the prevalence of asthma in childhood (58).

### Early Introduction of Fish in Diet

Among the studies analyzing the effect of an early introduction of fish on the development of respiratory outcomes, most of them (eight out of nine studies) suggest a protective role (Table 3).

A Dutch longitudinal study showed that introduction of fish between 6 and 12 months was associated with a lower prevalence of wheezing at the age of 2-year (59). In keeping with this, other studies demonstrated that fish consumption in the first year of life correlates with a reduced incidence of asthma and/or wheezing in preschool age (60–63) and up to the age of 12-year (64). Also, a recent study on 738 children showed that intake of all PUFAs in the first three years of life was inversely associated with asthma and/or recurrent wheezing, and this was statistically significant for DHA and linoleic acid (65).

On the other hand, one cohort prospective study (66) found no significant association between children's consumption of fish at 1 year of age and doctor-diagnosed asthma in subsequent years.

Taken together these studies suggest that introduction of fish in diet during the first year of life is protective toward wheezing and asthma, while the data on a possible beneficial effect of PUFA active supplementation on children's respiratory heath are more controversial.

## Vitamins A, C, and E

Vitamin A comes from plants (as carotenoids) or from animal-derived food sources (as retinol) and it has a major role in lung development, respiratory epithelium and immune system. World Health Organization (WHO) (67) recommends vitamin A supplementation for children above 6 months of age living in areas characterized by vitamin A deficiency.

Vitamin C, a water-soluble vitamin, has antioxidant capacity scavenging oxygen free radicals and suppressing macrophage secretion of superoxide anions (68). Vitamin E, a lipid-soluble vitamin, is the principal defense against oxidant-induced membrane injury. It also has non-antioxidant effect on immune functions (68).

Here we summarize the available trials which analyzed the role of vitamin A, vitamin C and vitamin E supplementations in the first year of life for the prevention of wheezing, asthma, and RTI.

### Vitamin A, C, and E in Prevention of Asthma and Wheeze

In the prevention of wheezing and asthma, few studies analyzed the impact of Vitamin A supplementation without unanimous results, while no studies have been published about vitamin C and E oral supplementations in the first year of life.

About Vitamin A, some authors described a higher prevalence of atopy and wheezing in children supplemented during the neonatal period with vitamin A, even if this effect is probably unrelated to later vitamin A status, influenced by selection bias and compromised by the high prevalence of drop-out during the 10 years of follow-up (69, 70). On the contrary, Kull et al. (41) demonstrated that supplementation, in the first year of life, of vitamin A and D in water-soluble form increased the risk of allergic disease up to the age of 4 years compared with supplementation with the same vitamins given in peanut oil.

### Vitamin A, C, and E in Prevention of RTI

Three trials (71–73), although without unanimous results, analyzed the effects of vitamin A supplementation in the first year of life on RTI, while no study evaluated the effect of vitamin C and E.

Long et al. (71) reported on 188 children, aged 6-15 months, assigned to receive vitamin A or placebo. Vitamin A supplementation reduced cough with fever but there were no significant differences in the incidence or duration of other types of RTI.

On the contrary, Tielsch et al. (72) enrolled 11,619 live-born infants to receive oral vitamin A or placebo following delivery and at 6 months of age, and showed in the vitamin A group a slightly higher rates for acute respiratory illness. Furthermore, Long et al. (73) in a double-blind, randomized, placebo-controlled trial in 736 children, aged 6–15 months, showed that Vitamin A supplementation was associated with a statistically significant increase (23%) in cough with fever.

## Conclusions

We reviewed the available scientific literature regarding early nutritional supplements in the prevention of wheezing, asthma, and RTI to understand if prebiotics and probiotics, vitamin D, PUFA, and vitamins A, C, and E could have a protective role.

As for pre- and probiotics, most of the studies showed no protective effect of early supplementation in the development of asthma and wheezing, while conflicting results were reported on their role in the reduction of RTI.

As for vitamin D, the available evidence suggests that early and regular (on a daily or weekly base) supplementation of vitamin D during infancy could have a role in the prevention of RTI, while most studies showed no protective effect toward wheezing or asthma development.

Finally, early introduction of fish in the diet seems protective toward wheezing and asthma development, while data on the effect of PUFA active supplementation on children's respiratory heath are more controversial.

## Author Contributions

VF, SC, and SZanc: conceptualization, methodology, and writing-review and editing. AT, SZane, RC, and FP: writing-original draft preparation. All authors contributed to the article and approved the submitted version.

## Conflict of Interest

The authors declare that the research was conducted in the absence of any commercial or financial relationships that could be construed as a potential conflict of interest.

## Publisher's Note

All claims expressed in this article are solely those of the authors and do not necessarily represent those of their affiliated organizations, or those of the publisher, the editors and the reviewers. Any product that may be evaluated in this article, or claim that may be made by its manufacturer, is not guaranteed or endorsed by the publisher.
